# Measurement uncertainty interval in case of a known relationship between precision and mean

**DOI:** 10.12688/f1000research.139111.1

**Published:** 2023-08-17

**Authors:** Steffen Uhlig, Bertrand Colson, Petra Gowik

**Affiliations:** 1 QuoData GmbH, Dresden, Saxony, 01309, Germany; 2 Federal Office of Consumer Protection and Food Safety, Berlin, 10117, Germany

**Keywords:** In-house validation study, reproducibility precision, measurement uncertainty, prediction interval, uncertainty interval

## Abstract

**Background:** Measurement uncertainty is typically expressed in terms of a symmetric interval *y±U*, where *y* denotes the measurement result and *U* the expanded uncertainty. However, in the case of heteroscedasticity, symmetric uncertainty intervals can be misleading. In this paper, a different approach for the calculation of uncertainty intervals is introduced.

**Methods:** This approach is applicable when a validation study has been conducted with samples with known concentrations. In a first step, test results are obtained at the different known concentration levels. Then, on the basis of precision estimates, a prediction range is calculated. The measurement uncertainty for a given test result can then be obtained by projecting the intersection of the test result with the limits of the prediction range back onto the axis of the known values, now interpreted as representing the measurand.

**Results:** It will be shown how, under certain circumstances, asymmetric uncertainty intervals arise quite naturally and lead to more reliable uncertainty intervals.

**Conclusions:** This article establishes a conceptual framework in which measurement uncertainty can be derived from precision whenever the relationship between the latter and concentration has been characterized. This approach is applicable for different types of distributions. Closed expressions for the limits of the uncertainty interval are provided for the simple case of normally distributed test results and constant relative standard deviation.

## Introduction

In this paper, a “top-down” approach for the calculation of measurement uncertainty is presented, in the sense that the estimate of measurement uncertainty is supported by precision data from a validation study.

Measurement uncertainty is defined in JCGM 100 as a parameter that “characterizes the dispersion of the values that could reasonably be attributed to the measurand”. ^1^ This parameter is often expressed as a standard deviation which is then used to obtain a symmetric uncertainty interval around the measurement result. In the following, the approach yielding symmetric uncertainty intervals will be referred to as the y±U approach.

As will be discussed below, in the case of heteroscedasticity, the y±U approach can yield misleading uncertainty intervals. For this reason, a different approach for determining measurement uncertainty is presented. This approach is suitable when precision data from a validation study conducted with test samples with *known* concentrations are available. While the focus in this paper is in-house validation, the approach presented here can also be applied for data from an interlaboratory validation study. It will be shown how, under certain circumstances, asymmetric uncertainty intervals arise quite naturally. For this reason, the approach presented here will be referred to in the following as the *asymmetric measurement uncertainty* approach (short form: *asymmetric* approach).

The asymmetric approach is perfectly consistent with the JCGM definition given above. Indeed, in the asymmetric approach, the focus is explicitly on the “dispersion of values which could reasonably be attributed to the measurand.” Furthermore, it draws the same distinction between measurand ( Y ) and measurement ( $Y_{m}$ ) as JCGM 106. ^2^ Even though the approach presented here is not Bayesian, there are important connections with JCGM 106, such as the definition of the best estimate of the measurand as $E\left(Y|Y_{m}\right)$ .

First, an experimental design for an in-house validation study and a statistical model are presented, allowing the calculation of in-house reproducibility precision as a function of concentration. Then it is shown how y±U uncertainty intervals are calculated from such precision data. The inconsistencies of the y±U approach are then discussed, and the asymmetric approach is presented. Throughout, the various concepts are illustrated with examples.

## Methods

### Experimental design and statistical model for an in-house validation study with samples with known concentrations

It is assumed that an in-house validation study has been conducted with samples with *known* concentrations. In the simplest case, the samples with known concentrations are obtained by diluting certified reference material. For each concentration level, several measurement results are obtained under in-house reproducibility conditions. This is best achieved via a factorial design, such as described in ISO/TS 23471. ^3^

The following table ( Table 1) provides an example of a factorial design with 7 factors and 8 factor level combinations.

**Table 1.** Factorial design with 7 factors, each with two levels.

Factor level combination j	Block (e.g. week)	Factors						
		1	2	3	4	5	6	7
**1**	**1**	1	1	1	2	2	2	1
**2**	**2**	1	1	2	2	1	1	2
**3**	**3**	1	2	1	1	2	1	2
**4**	**4**	1	2	2	1	1	2	1
**5**	**5**	2	1	1	1	1	2	2
**6**	**6**	2	1	2	1	2	1	1
**7**	**7**	2	2	1	2	1	1	1
**8**	**8**	2	2	2	2	2	2	2

More generally, the number of factor level combinations is denoted n . With m (known) concentration levels and p replicates per concentration level, a total of m∙p∙n measurements are performed.

The statistical model (mixed linear model) is as follows:

$$Y_{\mathrm{ijk}}=\alpha +\beta x_{\mathrm{ij}}+A\left(j\right)+B\left(j\right)x_{\mathrm{ij}}+A_{j}+B_{j}x_{\mathrm{ij}}+a_{\mathrm{ijk}}+b_{\mathrm{ijk}}x_{\mathrm{ij}}$$ (1)

where

$Y_{\mathrm{ijk}}$ denotes the measurement result at concentration level i=1,…,m for factor level combination j=1,…,n and replicate k=1,…,p

$x_{\mathrm{ij}}$ denotes the known concentration,

α and $\left(\beta -1\right)$ denote the absolute and the relative components of method bias. The α+β∙x curve represents the expected measured concentration at known concentration x and will be referred to in the following as the *mean curve.*

$A\left(j\right)$ and $B\left(j\right)$ denote the absolute and the relative components of the effect of factor level combination j (each of these terms results from summing effects of the individual factors).

$A_{j}$ and $B_{j}$ denote the absolute and the relative components of block effect j

$a_{\mathrm{ijk}}$ and $b_{\mathrm{ijk}}$ denote the absolute and the relative components of the repeatability error for measurement result $Y_{\mathrm{ijk}}$ .

In the following, two in-house validation data sets will be considered: Thiamphenicol in milk (Example 1) and Clopidol in egg (Example 2).

The following table ( Table 2) provide test results for Example 1 ( m=4 known concentration levels, p=1 replicate).

**Table 2.** Example 1 – design and test results from an in-house validation study for Thiamphenicol in milk.

Block j	Batch of milk	Storage	Technician	Mixer	CL 01 (25) [μg/kg]	CL 02 (50) [μg/kg]	CL 03 (75) [μg/kg]	CL 04 (100) [μg/kg]
01	Milk A	Storage A	Technician 1	Mixer A	23.9	51.9	74.9	100.9
02	Milk A	Storage A	Technician 2	Mixer B	24.3	50.5	74.2	99.3
03	Milk A	Storage B	Technician 1	Mixer B	24.8	49.9	73.6	97.6
04	Milk A	Storage B	Technician 2	Mixer A	29.2	55.3	79.4	102.7
05	Milk B	Storage B	Technician 2	Mixer B	28.4	53.4	78.3	103.1
06	Milk B	Storage B	Technician 1	Mixer A	26.5	51.3	77.9	101.8
07	Milk B	Storage A	Technician 2	Mixer A	25.0	52.9	77.2	102.1
08	Milk B	Storage A	Technician 1	Mixer B	25.5	51.7	74.3	98.4

**Figure 1.**
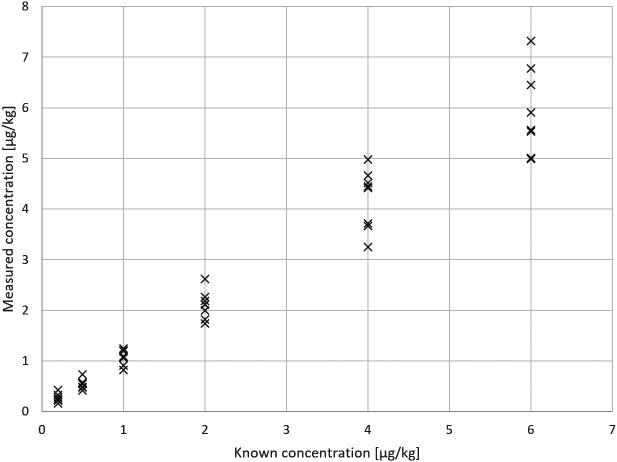
Example 1 - test results.

The test results for Example 1 are displayed in Figure 1.

The following table ( Table 3) provides test results for Example 2 ( m=6 known concentration levels, p=1 replicate).

**Table 3.** Example 2 – design and test results from an in-house validation study for Clopidol in egg.

Block j	Breeding	Operator	HPLC	Extract storage	CL 01 (0.2) [μg/kg]	CL 02 (0.5) [μg/kg]	CL 03 (1) [μg/kg]	CL 04 (2) [μg/kg]	CL 05 (4) [μg/kg]	CL 06 (6) [μg/kg]
01	Conventional	Routine	Batch 1 (Old)	With	0.22	0.49	0.82	2.11	4.66	6.45
02	Conventional	Routine	Batch 2 (New)	With	0.24	0.47	1.20	2.18	4.98	7.32
03	Conventional	Occasional	Batch 1 (Old)	Without	0.28	0.57	1.07	2.62	3.67	6.78
04	Conventional	Occasional	Batch 2 (New)	Without	0.22	0.55	1.06	1.74	3.72	5.56
05	Organic	Routine	Batch 1 (Old)	Without	0.16	0.42	0.91	2.00	4.42	5.53
06	Organic	Routine	Batch 2 (New)	Without	0.23	0.55	1.24	2.11	4.52	5.91
07	Organic	Occasional	Batch 1 (Old)	With	0.42	0.73	1.19	2.26	4.44	4.99
08	Organic	Occasional	Batch 2 (New)	With	0.32	0.56	1.08	1.82	3.25	5.01

**Figure 2.**
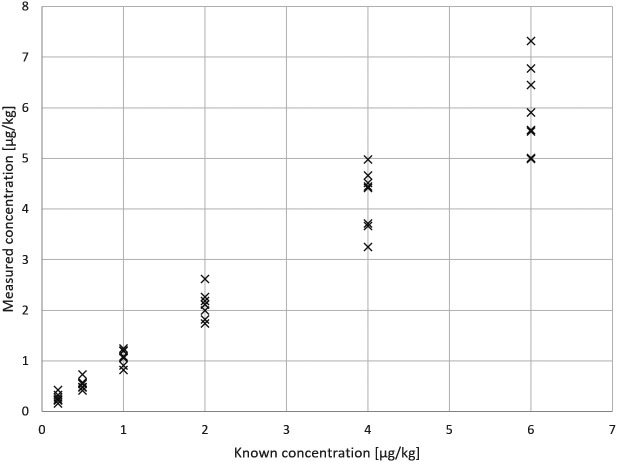
Example 2 - test results.

The test results for Example 2 are displayed in Figure 2.

For each example, reproducibility standard deviation values are calculated by means of Equation 1. With the exception of the two fixed effects α and β , the terms on the right side of Equation 1 are modelled as random variables. The corresponding variance components are denoted as follows:

$$A\left(j\right)=A_{1}\left(s_{1}\left(j\right)\right)+\cdots +A_{q}\left(s_{q}\left(j\right)\right)$$

$$B\left(j\right)=B_{1}\left(s_{1}\left(j\right)\right)+\cdots +B_{q}\left(s_{q}\left(j\right)\right)$$

where

$A_{1}\left(s\right),\ldots ,A_{q}\left(s\right)$ are random variables with zero mean and variances $\sigma_{A,1}^{2},\ldots ,\sigma_{A,q}^{2}$ , respectively

$B_{1}\left(s\right),\ldots ,B_{q}\left(s\right)$ are random variables with zero mean and variances $\sigma_{B,1}^{2},\ldots ,\sigma_{B,q}^{2}$ , respectively and where

$\left(s_{1}\left(j\right),\ldots ,s_{q}\left(j\right)\right)$ denotes the vector of factor levels for the q factors and for factor level combination j

Moreover,

$A_{1},\ldots ,A_{n}$ are random variables with zero mean and variance $\sigma_{A}^{2}$

$B_{1},\ldots ,B_{n}$ are random variables with zero mean and variance $\sigma_{B}^{2}$ .

$a_{111},\ldots ,a_{\mathrm{mnp}}$ are random variables with zero mean and variance $\sigma_{a}^{2}$

$b_{111},\ldots ,b_{\mathrm{mnp}}$ are random variables with zero mean and variance $\sigma_{b}^{2}$

The estimation of variance components in mixed linear models is described, for example, in Searle *et al.*, ^4^ McCulloch *et al.* ^5^ and Clarke. ^6^ Estimates for Example 1 are provided Table 4.

**Table 4.** Example 1 – variance estimates.

Variance component	Constant (A)	Proportional (B)
Repeatability	0.90760	0.00000
Block	0.88789	0.00000
Factor: Batch	0.00000	0.00004
Factor: Storage	1.06201	0.00000
Factor: Technician	1.52630	0.00000
Factor: Mixer	0.00000	0.00029

The in-house precision parameters for Example 1 for four different concentrations are provided in Table 5. At a given known concentration x , the in-house reproducibility standard deviation is calculated as follows:

$$\sigma_{\mathrm{Ri}}^{2}\left(x\right)=\sigma_{A,1}^{2}+\ldots +\sigma_{A,q}^{2}+x^{2}\left(\sigma_{B,1}^{2}+\ldots +\sigma_{B,q}^{2}\right)+\sigma_{A}^{2}+x^{2}\sigma_{B}^{2}+\sigma_{a}^{2}+x^{2}\sigma_{b}^{2}.$$ (2)

**Table 5.** Example 1 – In-house precision estimates.

Concentration [μg/kg]	In-house repeatability [%]	Block [%]	Factors [%]	In-house reproducibility [%]	In-house reproducibility [μg/kg]
25	3.8	3.8	6.7	8.6	2.14
50	1.9	1.9	3.7	4.6	2.28
75	1.3	1.3	2.8	3.3	2.50
100	1.0	0.9	2.4	2.8	2.77

The following two tables ( Table 6 & Table 7) provide variance and precision estimates for Example 2.

**Table 6.** Example 2 – variance estimates.

Variance component	Constant (A)	Proportional (B)
Repeatability	0.00000	0.01096
Block	0.00142	0.00524
Factor: Breeding	0.00118	0.00048
Factor: Operator	0.00749	0.00447
Factor: HPLC	0.00000	0.00000
Factor: Extract storage	0.00258	0.00000

**Table 7.** Example 2 – In-house precision estimates.

Concentration [μg/kg]	In-house repeatability [%]	Block [%]	Factors [%]	In-house reproducibility [%]	In-house reproducibility [μg/kg]
0.2	10.5	20.2	53.5	58.1	0.12
0.5	10.5	10.4	22.3	26.8	0.13
1	10.5	8.2	12.7	18.4	0.18
2	10.5	7.5	8.8	15.6	0.31
4	10.5	7.3	7.5	14.8	0.59
6	10.5	7.3	7.3	14.7	0.88

### Calculation of the y±U measurement uncertainty

The estimate of reproducibility at concentration x (see Equation 2) can be used to derive an estimate of the standard measurement uncertainty for the measurand Y as a function of x :

$$u\left(x\right)=\sqrt{\sigma_{\mathrm{Ri}}^{2}\left(x\right)}$$

The expanded measurement uncertainty is then obtained as follows:

$$U\left(x\right)=k∙u\left(x\right)$$

where k denotes the coverage factor.

It should be noted that it may be necessary to include the uncertainty of bias correction and the uncertainty of the (certified reference) values for the known concentrations used in the validation study, as appropriate. (see step 3 of Asymmetric measurement uncertainty approach).

Based on the precision values from the previous section, and using k=2 (for the sake of simplicity), one obtains the following expanded uncertainty values ( Table 8 & Table 9):

**Table 8.** Example 1 – Expanded uncertainty.

Concentration [μg/kg]	Expanded measurement uncertainty [%]	Expanded measurement uncertainty [μg/kg]
25	17.1	4.28
50	9.1	4.56
75	6.7	5.00
100	5.5	5.54

**Table 9.** Example 2 – Expanded uncertainty.

Concentration [μg/kg]	Expanded measurement uncertainty [%]	Expanded measurement uncertainty [μg/kg]
0.2	116.3%	0.23
0.5	53.6%	0.27
1	36.8%	0.37
2	31.2%	0.62
4	29.6%	1.19
5.5	29.4 %	1.62
6	29.3%	1.76

### The inconsistency of the *y* ± *U* approach in the case of heteroscedasticity

Typically, the expanded measurement uncertainty is used to construct a measurement uncertainty interval of the form y±U . Such symmetric measurement uncertainty intervals are not always appropriate. This will be demonstrated on the basis of a theoretical example.

In this example, it is assumed that the relative measurement uncertainty is *already known* and is constant at 35% across the applicable range of concentrations as specified in the scope of the analytical method. If the measurement result is y=10 (in this theoretical example, the unit plays no role and is therefore suppressed), then, applying the y±U approach with k=2 (for the sake of simplicity) and with the standard uncertainty obtained from the known 35% relative standard deviation (RSD) value, a measurement uncertainty interval of [3, 17] is obtained. According to the JCGM definition of measurement uncertainty discussed in the introduction, this means that the value y=3 could *reasonably be attributed to the measurand.* However, if the measurement result had been y=3 (instead of y=10 ), applying the same 35 % relative measurement uncertainty would result in an uncertainty interval of [0.9, 5.1]. Since the original value of y=10 does not lie within this second interval, it can be seen that the y±U approach is inconsistent in such a situation.

The two uncertainty intervals just discussed are summarized in the following table ( Table 10).

**Table 10.** Two y±U measurement uncertainty intervals in the case of a constant 35 % relative measurement uncertainty across the applicable range of concentrations. (In this theoretical example, the unit plays no role and is therefore suppressed.)

Lower limit	Measurement result	Upper limit
0.9	3	5.1
3	10	17

**Figure 3.**
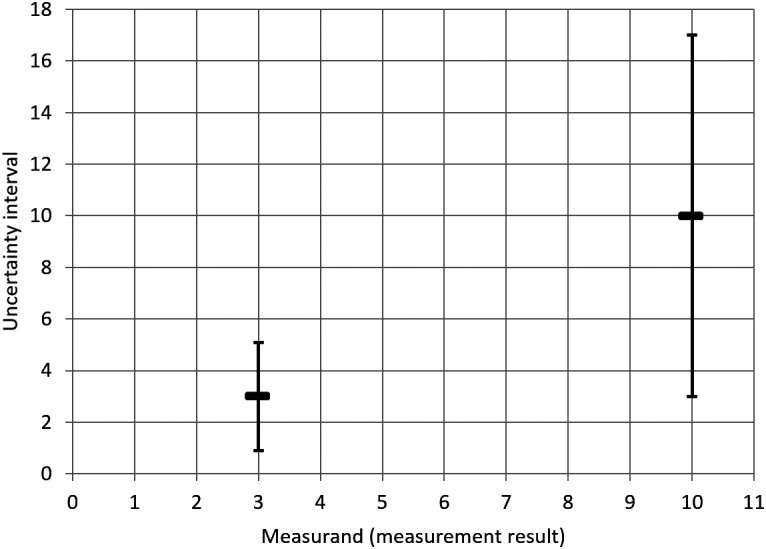
Uncertainty intervals (vertical axis) following the y±U approach for a constant RSD of 35%, for two measurement results (horizontal axis). (In this theoretical example, the unit plays no role and is therefore suppressed).

The inconsistency of the y±U approach discussed above is illustrated in the following figure ( Figure 3). Due to the constant RSD of 35 %, the width of the uncertainty interval (in the diagram: the height of the uncertainty interval) depends on the measurement result. Considered on its own, each uncertainty interval characterizes the dispersion of values that could be reasonably be attributed to the measurand on the basis of the measurement result. However, considered together, the different uncertainty intervals display inconsistencies. In particular, the value 10 does not lie within the uncertainty interval for the measurement result 3 , even though the latter lies within the uncertainty interval constructed around the former.

**Figure 4.**
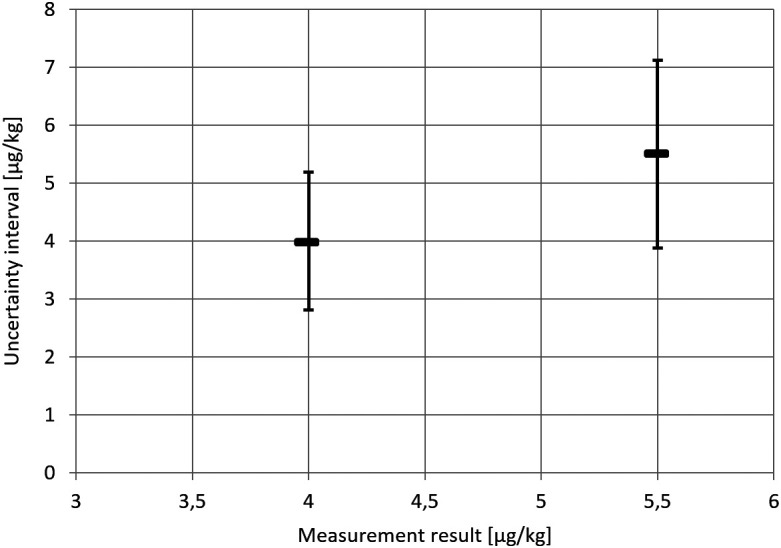
Uncertainty intervals (vertical axis) following the y±U approach on the basis of the data from Example 2 (Clopidol in egg) for two measurement results (horizontal axis). For a measurement result of 4 μg/kg (say in routine testing), values above 5.19 do not lie within the uncertainty interval. However, according to the evaluation of the data from the in-house validation study, measurement results ≤ 4 μg/kg were consistent with a measurand value of 5.5 μg/kg.

The inconsistency of the y±U approach will now be shown on the basis of the uncertainty intervals for Example 2 provided in Table 9. At a Clopidol concentration of 4 μg/kg Clopidol, the y±U approach yields the uncertainty interval y ± 1.19 μg/kg. In routine testing, if the measurement result is y=4 is obtained, it will be concluded that values above 5.19 μg/kg cannot reasonably be attributed to the measurand. However, from the same Table 9, we know that the lower limit of the uncertainty interval for a sample with 5.5 μg/kg Clopidol concentration is 3.88 μg/kg (or 3.98 μg/kg if bias is considered). In other words, we know that a measurand value of 5.5 μg/kg is perfectly compatible with a measurement result of 4 μg/kg. It follows that the value 5.5 μg/kg should lie within the measurement uncertainty interval for a measurement result of 4 μg/kg. This is not the case for the uncertainty interval obtained via the y±U approach. This is illustrated in the following figure ( Figure 4).

As has just been seen, in the presence of heteroscedasticity, the y±U approach leads to inconsistencies. For this reason, a different approach for the determination of measurement uncertainty in the case to heteroscedastic data is required. Such an approach will now be presented.

## Asymmetric measurement uncertainty approach

### Description of the approach

**Step 1: prediction range**

For each known concentration, the distribution of test results can be characterized in terms of a *prediction* interval. This interval reflects the degree to which the test results agree with one another, at the given concentration level and under the specified conditions (e.g. repeatability or in-house reproducibility). For a chosen prediction probability level (e.g. $p_{\text{pred}}=95\%$ ), a subsequent test result will lie inside the prediction interval with probability $p_{\text{pred}}$ .

**Figure 5.**
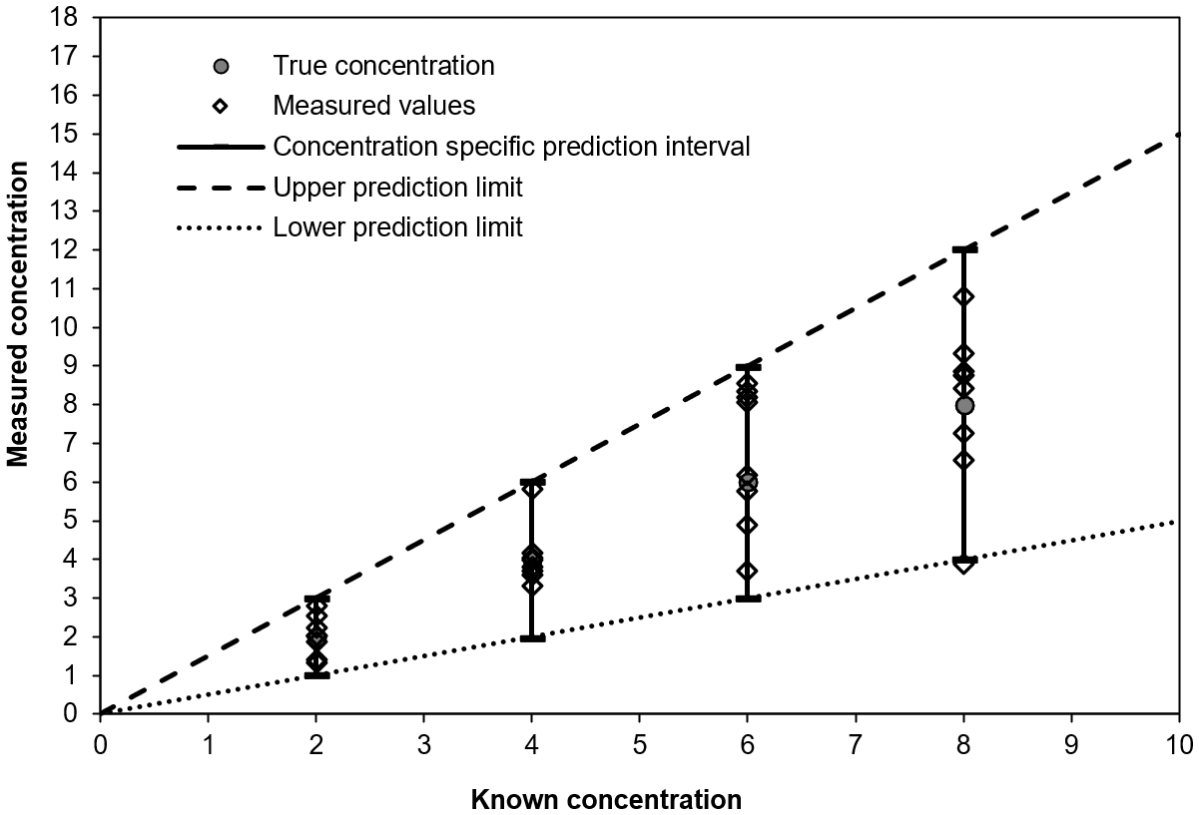
Construction of a prediction range. For each known concentration, the diamonds represent the measurement results and the solid vertical line represents the prediction interval. The prediction range is represented by the dashed lines.

If measurements are performed at different known concentrations, then it is possible to construct a prediction *range*, rather than individual prediction *intervals.* This step involves applying one statistical model to all the data, as described the previous section. The construction of a prediction range is described in the following figure ( Figure 5).

For further information regarding the computation of a prediction range, the reader is referred to the discussion of variance functions in ISO 5725-2 ^7^ and to the in-house validation approach described in Gowik *et al*. ^8^ and Jülicher *et al.* ^9^ [ 1].

**Step 2: measurement uncertainty interval**

Once a prediction range has been calculated, the measurement uncertainty interval for a given test results (obtained e.g. in routine testing) can be determined. Step 2 no longer involves the *data* from the validation study. Rather, for Step 2, it is assumed that a prediction range has previously been calculated and is thus available.

Accordingly, the meaning of the axes is now different. The vertical axis now represents the measurement result obtained, say, in routine testing and denoted $y_{m}$ , while the horizontal axis now represents the measurand (which is to be characterized via the measurement) denoted y (This notation is chosen so as to be consistent with JCGM 106, though, strictly speaking, $y_{m}$ is one realization of a random variable $Y_{m}$ and y is one realization of a random variable Y . However, a distinction between random variables and their realizations is not required in this paper.).

The starting point for Step 2 is a test result, displayed on the $y_{m}$ -axis. The intersection of the $y_{m}$ -value with the upper prediction curve is projected onto the y -axis to obtain the lower measurement uncertainty limit. Indeed, for measurand values below this y -value, measurement results can be expected to be less than the $y_{m}$ -value. Secondly, the intersection of the $y_{m}$ -value with the lower prediction curve is projected onto the y -axis to obtain the upper measurement uncertainty limit. Indeed, for a measurand values above this y -value, measurement results can be expected to be greater than the $y_{m}$ -value. The resulting y -axis interval (grey horizontal band hugging the y-axis) thus corresponds to the values which could “reasonably be attributed to the measurand.”

**Figure 6.**
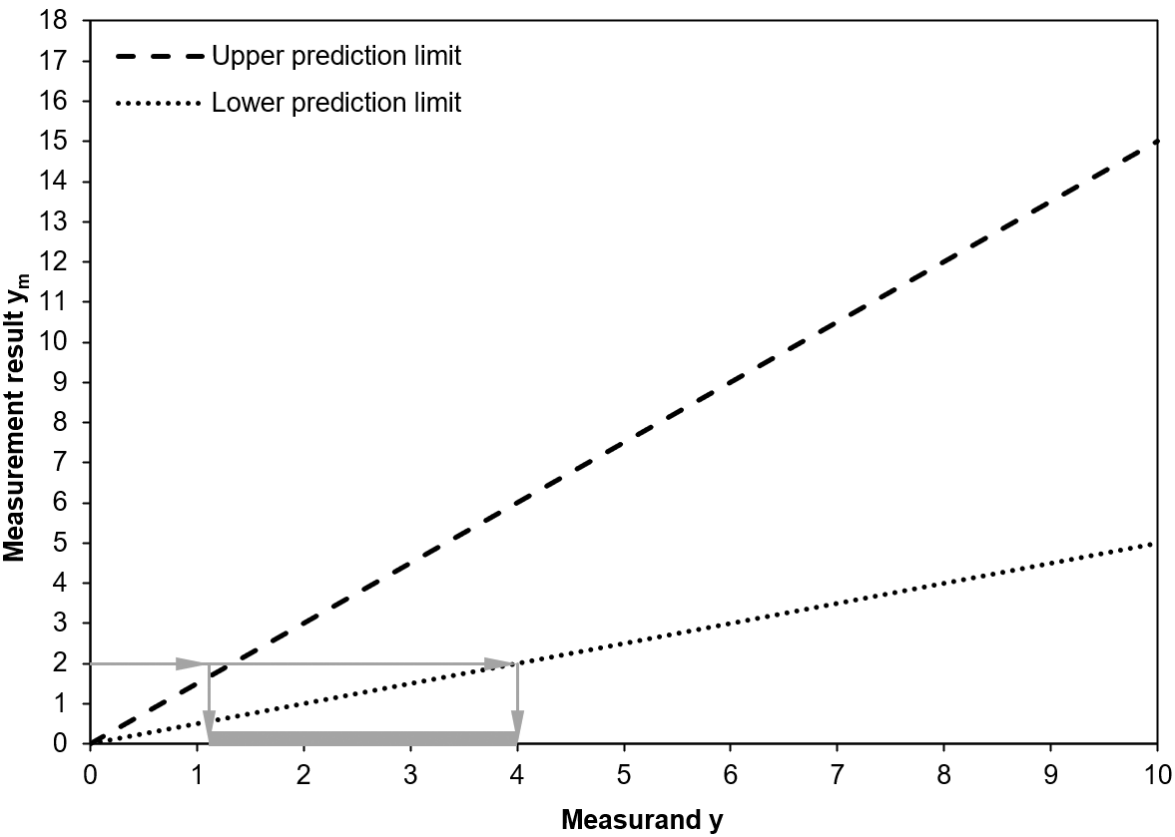
Procedure for obtaining a measurement uncertainty interval from a measurement result.

This procedure is illustrated in Figure 6.

**Step 3: best estimate for the measurand**

The “best estimate of the measurand” is the projection onto the y -axis of the intersection of the measurement result ( $y_{m}$ -value) with the mean curve α+β∙y (see previous section with the measurand y replacing the known concentration x ). If the bias is negligible (i.e. α is close to zero and β is close to 1), then this y -value will be close to the $y_{m}$ -value. However, if a bias is present, then the two values will differ, and taking the y -axis projection corresponds to a bias or recovery correction. For this reason, the y -axis projection is denoted $y_{\text{corr}}$ . If such a bias or recovery correction is performed, it is recommended to include the “uncertainty of the bias correction” (see Ref. 1) as an additional variance component in the calculation of the prediction intervals at each known concentration x during the validation study. The uncertainty of bias correction, in turn, may consist of various sources of uncertainty such as the statistical uncertainty of the parameters α and β and the uncertainty of reference values used as *known concentrations.*

Finally, it should be noted that the symmetry of the measurement uncertainty interval is determined in relation to $y_{\text{corr}}$ .

### Calculation of the measurement uncertainty intervals

In this section, it is shown how to calculate the lower and upper limits of asymmetric measurement uncertainty intervals.

It is assumed that there is no bias and that, for each concentration level in the recovery experiment, the measurement results follow a normal distribution.

Let $y_{L}$ denote the lower limit of the expanded uncertainty interval and $y_{U}$ the corresponding upper limit. Furthermore, let $f_{U}\left(y\right)$ denote the upper limit of the prediction interval at measurand value y , and let $f_{L}\left(y\right)$ denote the lower limit of the prediction interval at y . For a given measurement result $y_{m}$ , the two limits $y_{L}$ and $y_{U}$ can then be computed iteratively, using the implicit relationships

$$f_{U}\left(y_{L}\right)=y_{m}$$ (3)

$$f_{L}\left(y_{U}\right)=y_{m}$$ (4)

In the case of a constant relative standard deviation $\sigma_{\mathrm{rel}}$ across all concentration levels, and in the absence of bias, explicit expressions for $y_{L}$ and $y_{U}$ can be provided. Indeed, in such a situation, a given measurement result $y_{m}$ obtained to characterize the (unknown) measurand y will lie with 95 % probability [ 2] in the interval $\left[y-2∙y∙\sigma_{\mathrm{rel}},y+2∙y∙\sigma_{\mathrm{rel}}\;\right]$ , i.e.

$$y-2∙y∙\sigma_{\mathrm{rel}}\leq y_{m}\leq y+2∙y∙\sigma_{\mathrm{rel}}$$ (5)

The above inequality can be rewritten as

$$\begin{array}{c} y∙\left(1-2∙\sigma_{\mathrm{rel}}\right)\leq y_{m}\leq y∙\left(1+2∙\sigma_{\mathrm{rel}}\right)\\ ⟺\frac{1-2∙\sigma_{\mathrm{rel}}}{y_{m}}\leq \frac{1}{y}\leq \frac{1+2∙\sigma_{\mathrm{rel}}}{y_{m}}\\ ⟺\frac{y_{m}}{1+2∙\sigma_{\mathrm{rel}}}\leq y\leq \frac{y_{m}}{1-2∙\sigma_{\mathrm{rel}}} \end{array}$$ (6)

Thus, the measurand y will lie in the interval $\left[\frac{y_{m}}{1+2∙\sigma_{\mathrm{rel}}},\frac{y_{m}}{1-2∙\sigma_{\mathrm{rel}}}\right]$ with 95% probability.

For example, for a constant relative standard deviation of 40% (i.e. $\sigma_{\mathrm{rel}}=0.4$ ) and for the measurement result $y_{m}=100$ , the lower and upper uncertainty limits are computed as:

$$y_{L}=\frac{100}{1.8}\approx 56$$

$$y_{U}=\frac{100}{0.2}=500$$

As long as the exact quantile is used and the assumptions are valid, this uncertainty interval is statistically exact in the sense that the coverage probability is exactly 95%.

### Symmetric versus asymmetric intervals

**When is the proposed uncertainty interval symmetric?**

**Figure 7.**
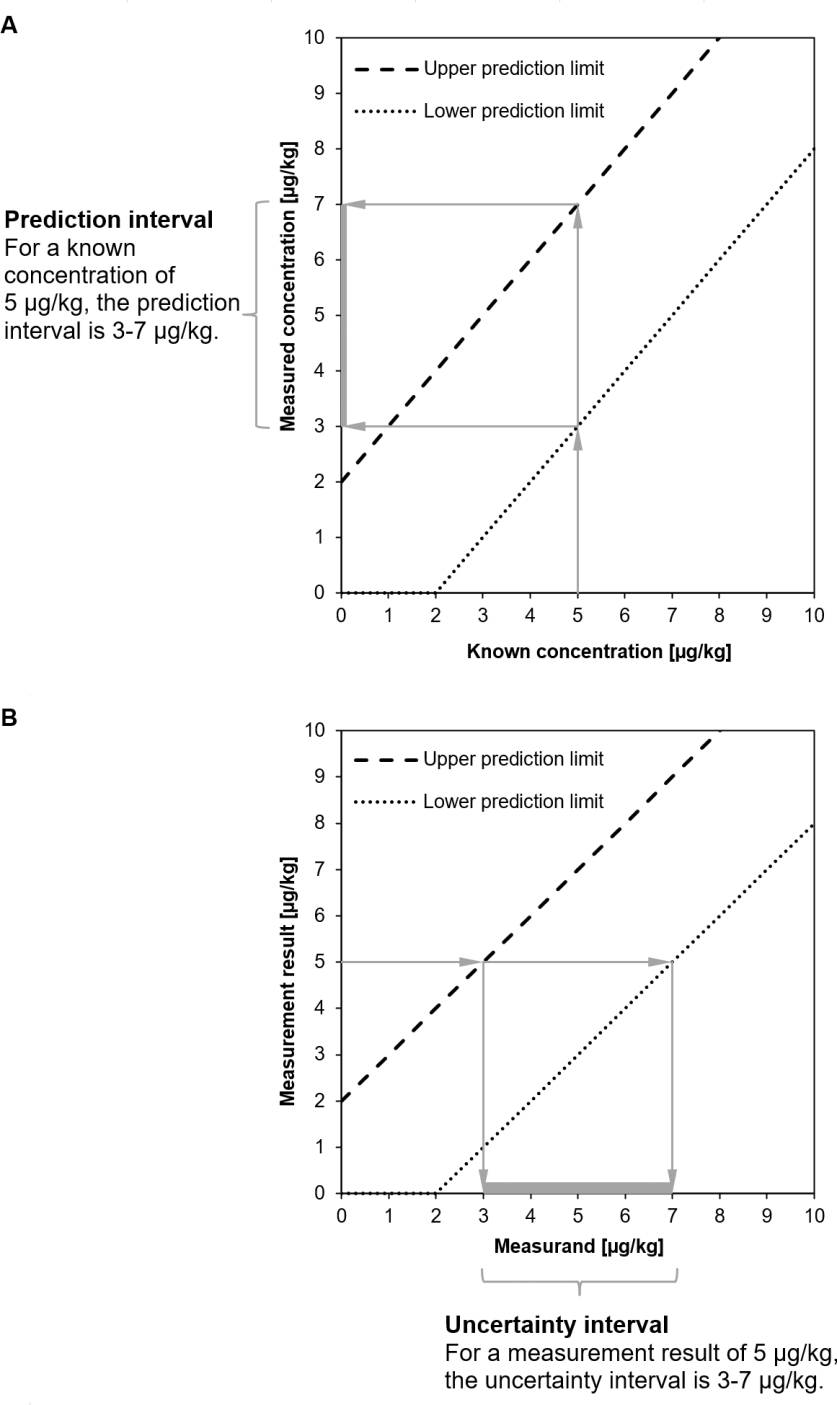
Uncertainty intervals (A) following the y±U approach and (B) following the asymmetric approach. If there is no bias and if the prediction limits run parallel to the mean curve α+β∙x , then the intervals obtained from the two approaches are identical.

If the prediction limits run parallel to the mean curve α+β∙x (see Section 0), the uncertainty interval is perfectly symmetric. This is the case when it is the absolute rather than the relative standard deviation which is constant across concentration levels. It should also be noted that, when the prediction limits run parallel to the mean curve α+β∙x and when there is no bias (100 % recovery), then the prediction and measurement uncertainty intervals are identical. This is illustrated in the following figure ( Figure 7).

The assumption that an uncertainty interval is symmetric is justified if the following two conditions are met:

Condition 1: Measurement results are distributed symmetrically around the corresponding mean value at a given known concentration in the validation study.

Condition 2: Heteroscedasticity is negligible.

**When is the proposed uncertainty interval asymmetric?**

In the field of analytical chemistry, in most cases, the distribution of results obtained from measurements performed on one and the same sample is more or less symmetric, so that symmetry condition 1 above is met. Condition 2, however, is almost never met. In the case of weakly heterogeneous variances, uncertainty intervals may still be *approximately* symmetric. Similarly, if the spread of measurement results at any given concentration level remains small (i.e. the relative standard deviations are small), uncertainty intervals may still be *approximately* symmetric. However, if the dispersions vary considerably across concentration levels *and* the corresponding relative standard deviations are large (say, greater than 10 %), then it is necessary to take the asymmetry of the uncertainty intervals into account. Restricting the concentration range under consideration in order to avoid variance heterogeneity is common analytical practice. In some cases, this expedient may allow the symmetry assumption to be applied.

**Figure 8.**
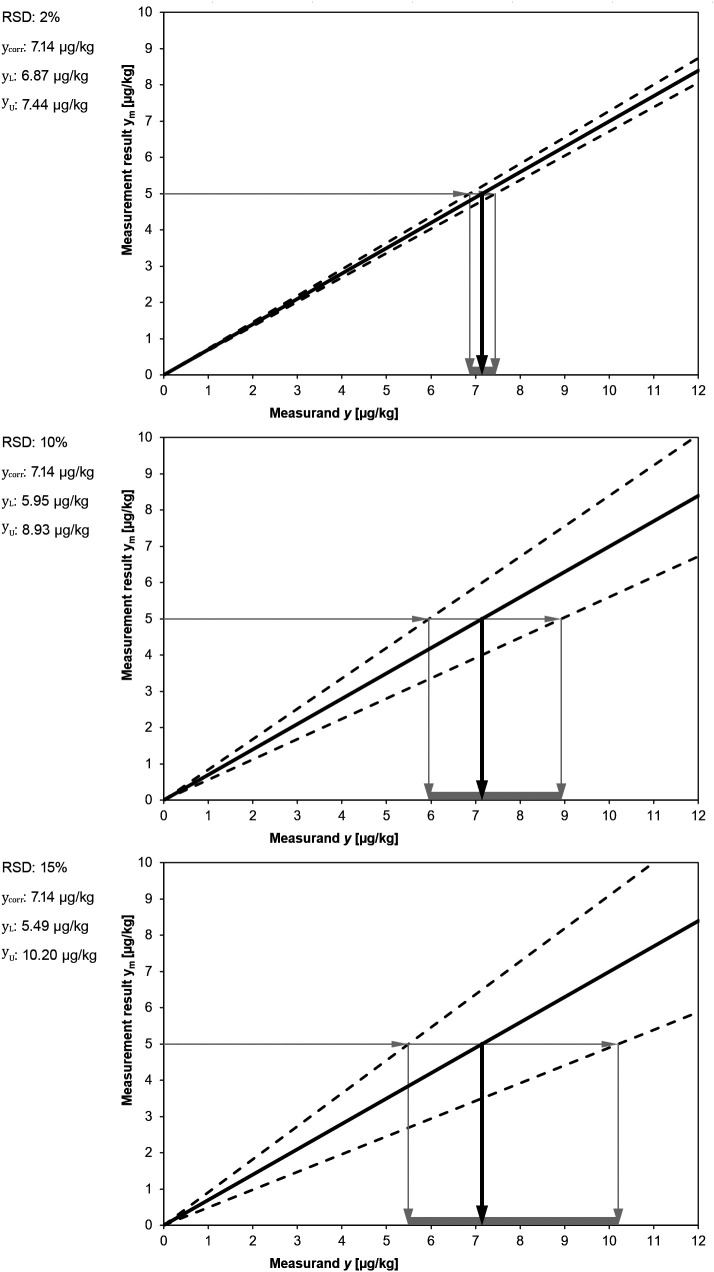
Measurement uncertainty intervals constructed following the asymmetric approach, starting from the same measurement result $y_{m}=5$ . The degree of asymmetry depends on the magnitude of the variance.

The following figure ( Figure 8) illustrates the relationship between magnitude of dispersion and symmetry of the uncertainty interval.

## Applying the alternative approach to the data from the factorial in-house validation studies (Example 1 and Example 2)

The uncertainty intervals for Example 1 and Example 2 are provided in the following tables ( Table 11 & Table 12). The degree of asymmetry of a given uncertainty interval can be gauged by comparing the values of the differences $y_{\text{corr}}-y_{L}$ and $y_{U}-y_{\text{corr}}$ (See earlier sections for the notation $y_{L}$ , $y_{U}$ and $y_{\text{corr}}$ .). The two values $y_{\text{corr}}-y_{L}$ and $y_{U}-y_{\text{corr}}$ can also be compared with the expanded uncertainty from the y±U approach. Accordingly, the U values from Table 8 and Table 9 are reproduced here for convenient reference.

**Table 11.** Example 1 – Lower and upper limits of the uncertainty intervals.

Measured concentration [μg/kg] $y_{m}$	Lower limit $y_{L}$	Upper limit $y_{U}$	Best estimate of measurand $y_{\text{corr}}$	Difference $y_{\text{corr}}-y_{L}$	Difference $y_{U}-y_{\text{corr}}$	Expanded uncertainty for y±U U
25	19.2	27.8	23.51	4.31	4.29	4.28
50	44.2	53.3	48.66	4.46	4.64	4.56
75	68.9	78.9	73.81	4.91	5.09	5.00
100	93.5	104.7	98.97	5.47	5.73	5.54

**Table 12.** Example 2 – Lower and upper limits of the uncertainty intervals.

Measured concentration [μg/kg] $y_{m}$	Lower limit $y_{L}$	Upper limit $y_{U}$	Best estimate of measurand $y_{\text{corr}}$	Difference $y_{\text{corr}}-y_{L}$	Difference $y_{U}-y_{\text{corr}}$	Expanded uncertainty for y±U U
0.2	0.00	0.39	0.14	0.14	0.25	0.23
0.5	0.21	0.75	0.44	0.23	0.31	0.27
1	0.65	1.40	0.94	0.29	0.46	0.37
2	1.45	2.76	1.93	0.48	0.83	0.62
4	3.02	5.53	3.91	0.90	1.62	1.19
6	4.57	8.31	5.90	1.34	2.41	1.76

As can be seen, for Example 1, the values for the differences $y_{\text{corr}}-y_{L}$ and $y_{U}-y_{\text{corr}}$ lie relatively close to one another and to the U values. By contrast, for Example 2, the difference values differ considerably from one another and from the U values. For instance, for $y_{m}=2$ μg/kg, we have

$$y_{\text{corr}}-y_{L}=0.48\;\mu \mathrm{g/kg}$$

$$y_{U}-y_{\text{corr}}=0.83\;\mu \mathrm{g/kg}$$

while

U=0.62μg/kg.

**Figure 9.**
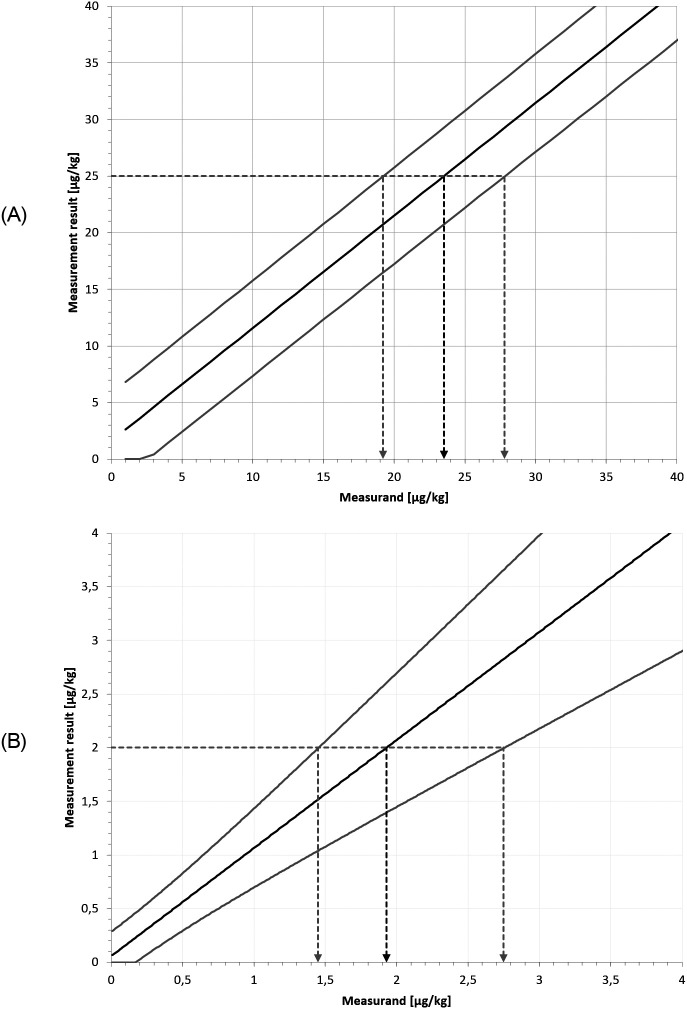
Calculation of uncertainty limits and best measurand values for (A) Example 1 and (B) Example 2.

The calculation of the upper and lower limits, as well as the best estimate of the measurand $y_{\text{corr}}$ is illustrated in the following figure ( Figure 9).

## Conclusions

As has been seen, asymmetric measurement uncertainty intervals arise naturally in the case of heteroscedasticity. Accordingly, in such cases, the symmetric uncertainty interval y±U should be seen as a mere *approximation* of the exact measurement uncertainty interval. This approximation is adequate if variability is low (say, less than 10 % RSD). In the case of both high variability and heteroscedasticity, symmetric measurement uncertainty intervals can be misleading. An awareness of these issues is thus particularly important in fields where these two conditions are expected, such as chemical trace analysis.

In the examples considered in this paper, it is assumed that data follow a normal distribution. If data follow another distribution, the uncertainty interval is different. Lognormal data are a familiar case in point. Indeed, consider the case that for any given concentration x , the log-transformed test results can be expected to lie between $\ln\left(x\right)-2\sigma$ and $\ln\left(x\right)+2\sigma$ . Then, in the original domain (i.e. prior to the log-transformation), the RSD will remain constant across concentration levels and the dispersion will thus incre ase monotonically with the concentration.

In many cases a log-transformation stabilizes the variance, meaning that, in the log domain, all the data are normally distributed with one and the same standard deviation σ , independently of the concentration. Back-transformation (“anti-log”) then again provides asymmetric uncertainty intervals, from $y/\exp\left(\mathrm{k\sigma}\right)$ to $y∙\exp\left(\mathrm{k\sigma}\right)$ . These intervals are asymmetric, but to a lesser extent that the interval derived under the assumption that the original data follow a normal distribution. Take the case σ=RSD=0.25 and k=2 . Then the asymmetric uncertainty interval based on the assumption that the original data follow a normal distribution (see Equation 5) is $\left[\frac{y}{1.5},2y\right]$ , and the asymmetric uncertainty interval based on the log-normal distribution is $\left[\frac{y}{1.65},1.65y\right]$ .

However, a log-transformation does not always have this variance stabilization effect, even if the data follow a lognormal distribution. For instance, if the standard deviations in the original domain are constant across concentrations, then, in the log domain, the σ value will depend on the concentration.

More generally, this article establishes a conceptual framework in which measurement uncertainty can be derived from precision whenever the relationship between the latter and concentration has been characterized. Closed expressions for the limits of the uncertainty interval were provided for the simple case of normally distributed test results and constant relative standard deviation. In more complex cases, it may not be possible to provide closed expressions. However, iterative calculation procedures can be applied, and further work may be required to illustrate appropriate context-specific approaches.

## Data Availability

No other data were used than those provided in Table 2 and Table 3.

## Footnotes

^1^
This in-house validation approach is referenced in CD 657 ^10^ and CIR 808. ^11^

^2^
For the sake of legibility, the exact 95 % quantile has been replaced by the value 2.
