# Supplementary material for: Measurement uncertainty interval in case of a known relationship between precision and mean
Source: F1000Res. 2023 Aug 17;12:996. [Version 1] doi: 10.12688/f1000research.139111.1 (PMC10808853; doi:10.12688/f1000research.139111.1)
Supplement: Peer review history

# Reviewer report: Reviewer response for version 1

**DOI:** 10.5256/f1000research.152356.r232320

**Published:** 2024-02-20

**Authors:** Elcio Cruz de Oliveira

**Affiliations:** 1 Pontifical Catholic University of Rio de Janeiro, Rio de Janeiro, Brazil

This manuscript aimed to present a different approach for the calculation of uncertainty intervals in the case of heteroscedasticity. However, it is not clear in the abstract.

 After reading the entire document, I have some major comments:

 1 – I suggest that the keywords be placed in alphabetical order.

 2 – Introduction: Please clarify the relationship between “heteroscedasticity” and “asymmetric uncertainty intervals”.

 3 – Table 1. Factorial design with 7 factors, each with two levels. Did the authors use a Plackett–Burman design? Please clarify it better, explaining the reason and inserting more references.

 4 – Table 2. Example 1 – design and test results from an in-house validation study for Thiamphenicol in milk. It seems that the authors used a fractional factorial design, 2 ^4-1^, resolution IV. If I am correct, I did not understand the correlation between Table 2 and Table 1. Please, explain it better.

 5 – Eqs. (1) and (2): Do the data present homoscedastic or heteroscedastic behavior? Did the authors use a weighted linear regression or an unweighted linear regression? See references: Farias et al (2015) [Ref-1] and Caldeira et al (2017) [Ref-2]

 6 – Page 5: “… the in-house reproducibility…”, this term is not introduced in JCGM 200:2012. I assume the authors mean to refer to “intermediate precision”.

 7 – I recommend that authors include the Monte Carlo Method (MCM) to validate their study.

 8 – Conclusions should be more succinct and objective and future work requires greater clarification.

Is the work clearly and accurately presented and does it cite the current literature?

Yes

If applicable, is the statistical analysis and its interpretation appropriate?

Partly

Are all the source data underlying the results available to ensure full reproducibility?

Partly

Is the study design appropriate and is the work technically sound?

Partly

Are the conclusions drawn adequately supported by the results?

Partly

Are sufficient details of methods and analysis provided to allow replication by others?

Partly

Reviewer Expertise:

Chemical Metrology, focusing in measurement uncertainty, measurement uncertainty arising from sampling and use of uncertainty information in compliance assessment.

I confirm that I have read this submission and believe that I have an appropriate level of expertise to confirm that it is of an acceptable scientific standard, however I have significant reservations, as outlined above.

## References

[1] : METROLOGICAL EVALUATION OF THE ANALYTICAL CURVE FOR DETERMINATION OF VERY LOW CONCENTRATIONS OF SULPHUR CONTENT IN DIESEL OIL S-10. *Química Nova* .2015; 10.5935/0100-4042.20150071 10.5935/0100-4042.20150071

[2] : LC–MS/MS quantification of ractopamine in bovine and swine muscle: stability of matrix-matched calibration solutions. *Accreditation and Quality Assurance* .2017;22(2) : 10.1007/s00769-017-1255-y 91-96 10.1007/s00769-017-1255-y

# Reviewer report: Reviewer response for version 1

**DOI:** 10.5256/f1000research.152356.r232361

**Published:** 2024-01-24

**Authors:** Hariyanto Gunawan

**Affiliations:** 1 Chung Yuan Christian University,, Taoyuan, Taiwan

The authors introduced a different approach for the calculation of uncertainty intervals. This is an interesting topic due to the existence of uncertainty in measurement. However, I have some comments as follows:

 1. Give the full name of the abbreviation JCGM in the beginning.

 2. In Figure 1, the value of the horizontal and vertical axis must show the same number as in Table 2. Similar for Figure 2 and Table 3.

 3. The author should add a state of the art so the readers can understand the recent related research.

 4. In Figure 5, add units for the horizontal and vertical axis.  

 5. The author should directly write Table 1 instead of using the following Table (Table 1). Same for other tables and figures.

Is the work clearly and accurately presented and does it cite the current literature?

Yes

If applicable, is the statistical analysis and its interpretation appropriate?

Yes

Are all the source data underlying the results available to ensure full reproducibility?

No source data required

Is the study design appropriate and is the work technically sound?

Yes

Are the conclusions drawn adequately supported by the results?

Yes

Are sufficient details of methods and analysis provided to allow replication by others?

Yes

Reviewer Expertise:

Intelligent manufacturing system platform development, Artificial Intelligence, Internet of Things, Big data analysis, Industry 4.0 applications, real time monitoring.

I confirm that I have read this submission and believe that I have an appropriate level of expertise to confirm that it is of an acceptable scientific standard.

# Reviewer report: Reviewer response for version 1

**DOI:** 10.5256/f1000research.152356.r232307

**Published:** 2024-01-17

**Authors:** Chong Wang

**Affiliations:** 1 Beihang University, Beijing, China

This paper proposes a analysis framework to predict the measurement uncertainty interval in case of a known relationship between precision and mean. The research topic is interesting, and some minor comments are given as follows.

 1. Many abbreviations and mathematical symbols have been used in the manuscript. For better understanding to the readers, a nomenclature presented in tabular form is suggested.

 2. If possible, some existing methods for interval uncertainty prediction are suggested for comparison to verify the proposed method.

 3. Some of the figures, e.g. Fig.5, can be further improved to present the results more clearly.

Is the work clearly and accurately presented and does it cite the current literature?

Yes

If applicable, is the statistical analysis and its interpretation appropriate?

Yes

Are all the source data underlying the results available to ensure full reproducibility?

Yes

Is the study design appropriate and is the work technically sound?

Yes

Are the conclusions drawn adequately supported by the results?

Yes

Are sufficient details of methods and analysis provided to allow replication by others?

Yes

Reviewer Expertise:

uncertainty quantification and propagation

I confirm that I have read this submission and believe that I have an appropriate level of expertise to confirm that it is of an acceptable scientific standard.

# Reviewer report: Reviewer response for version 1

**DOI:** 10.5256/f1000research.152356.r197911

**Published:** 2023-11-13

**Authors:** Bertil Magnusson

**Affiliations:** 1 Trollboken, Göteborg, Sweden

The article introduces an important issue that a high relative uncertainties the uncertainty interval is not symmetric but asymmetric. It is well written and clear. The examples are easy to follow. 

 Some minor comments:

 1 The conclusion as stated above is missing in the abstract In the article.

 2 It would be helpful for the reader if constant RSD, which is very common for instrumental techniques at higher concentrations,  is described as one form of heteroscedasticity.

 3 The very important issue if uncertainty is related to the measured value or to the true value is just mentioned briefly and could have been expanded - it is important when the RSD is constant.

 "Furthermore, it draws the same distinction between measurand (YY) and measurement (YmYm) as JCGM 106. ^2^ Even though the approach presented here is not Bayesian, there are important connections with JCGM 106, such as the definition of the best estimate of the measurand as E(Y∣∣Ym)EYYm."

 4 The value of 10 % RSD is given without any examples for the reader to understand when it is critical ."However, if the dispersions vary considerably across concentration levels *and* the corresponding relative standard deviations are large (say, greater than 10 %), then it is necessary to take the asymmetry of the uncertainty intervals into account."

 5 Table captions should be easy to read without referring to much to the text - now just example 1 and 2. One is low and one is higher RSD

Is the work clearly and accurately presented and does it cite the current literature?

Yes

If applicable, is the statistical analysis and its interpretation appropriate?

Yes

Are all the source data underlying the results available to ensure full reproducibility?

Yes

Is the study design appropriate and is the work technically sound?

Yes

Are the conclusions drawn adequately supported by the results?

Yes

Are sufficient details of methods and analysis provided to allow replication by others?

Yes

Reviewer Expertise:

My area of research is quality if measurement . e.g. QC, validation und uncertainty and quality of decision on a result comparing with a specification

I confirm that I have read this submission and believe that I have an appropriate level of expertise to confirm that it is of an acceptable scientific standard, however I have significant reservations, as outlined above.
